# Improvement in Pulmonary Function Tests After Septoplasty: A Longitudinal Study

**DOI:** 10.7759/cureus.65492

**Published:** 2024-07-27

**Authors:** Mayur Ingale, Yash Kalra, Vinod Shinde, Apurva Jarandikar, Rashmi P Rajashekhar

**Affiliations:** 1 Department of Otolaryngology, Head and Neck Surgery, Dr. D. Y. Patil Medical College, Hospital and Research Centre, Dr. D. Y. Patil Vidyapeeth, Pune (Deemed to be University), Pune, IND

**Keywords:** snot 22 questionnaire, 6-minute walk test, pulmonary function test, deviated nasal septum, septoplasty

## Abstract

Introduction

The relationship between the nasal and pulmonary systems is rooted in the shared anatomy and physiology of the upper and lower respiratory tracts. Our study objective was to assess the improvement in pulmonary function tests (PFTs) after septoplasty in patients with a deviated nasal septum (DNS).

Methods

A longitudinal study was conducted at a tertiary care center from October 1, 2022, to March 31, 2024. Patients aged 18-55 years with chronic nasal obstruction due to an isolated DNS were included in the study. Patients under 18 or over 55 years of age, those undergoing combined nasal surgeries, and those with comorbidities such as hypertension, diabetes, chronic smoking, chronic obstructive pulmonary disease, bronchial asthma, turbinate hypertrophy, chronic sinusitis, or nasal polyposis were excluded. Pre-operative assessments included detailed ear, nose, and throat examinations, routine blood investigations, X-rays of the chest and paranasal sinuses (Waters' view), PFTs (spirometry), the Sino-Nasal Outcome Test-22 (SNOT22) questionnaire, and the six-minute walk test (6MWT). Post-operative assessments included repeated spirometry, a 6MWT at three weeks post-surgery, and the SNOT22 questionnaire for subjective symptom assessment.

Results

Participants included 30 males and 30 females, with a mean age of 35.6 ± 8.2 years. Significant improvements (p < 0.05) were observed in PFT parameters (forced expiratory volume in one second (FEV1), forced vital capacity (FVC), FEV1/FVC ratio, peak expiratory flow), exercise capacity (6MWT distance), and symptom severity (SNOT22 scores) post-septoplasty. High levels of patient satisfaction and notable improvements in quality of life were reported. The average hospital stay was 2.5 days.

Conclusion

Septoplasty in patients with DNS significantly improves pulmonary function, exercise capacity, and symptom severity, with high patient satisfaction and minimal complications.

## Introduction

The relationship between the upper and lower respiratory tracts has long been a subject of scientific investigation, with various studies exploring the potential impact of nasal anatomy on pulmonary function. One common structural anomaly that has garnered attention in this view is nasal septal deviation (NSD), a condition characterized by the displacement of the nasal septum from its central position. Septal deviation can impede normal nasal airflow, potentially influencing respiratory dynamics and contributing to a range of symptoms, from nasal congestion to respiratory distress [1].

Pulmonary function tests (PFTs) serve as invaluable tools for quantifying respiratory function and detecting abnormalities within the respiratory system. These tests cover a variety of measurements, including spirometry, lung volumes, and diffusion capacity, providing a comprehensive assessment of the efficiency with which the respiratory system exchanges gases. As researchers focus deeper into the connections between nasal and pulmonary physiology, questions arise regarding the potential benefits of surgical interventions, such as septoplasty, in improving pulmonary function in individuals with NSD [2].

NSD is a prevalent condition, affecting individuals across various age groups and demographics. The nasal septum, a vital structural component, separates the left and right nasal cavities. When deviated, it can lead to asymmetry in nasal airflow, compromising the efficiency of the respiratory process. While septal deviation is often associated with nasal symptoms such as congestion, rhinorrhea, and headaches, recent studies have begun to explore its potential implications for the lower respiratory system [3].

The relationship between the nasal and pulmonary systems is rooted in the shared anatomy and physiology of the upper and lower respiratory tracts. The nasal passages serve as the primary entry point for inspired air, where it undergoes filtration, humidification, and temperature regulation before reaching the lower respiratory tract. Any disruption in this process, such as that caused by NSD, may alter the dynamics of airflow, potentially impacting pulmonary function [4].

Existing literature has provided insights into the complex relationship between nasal and pulmonary physiology. Studies have shown that nasal obstruction, a common consequence of septal deviation, can lead to changes in breathing patterns, with individuals adopting compensatory mechanisms to maintain adequate airflow. These adaptations may involve alterations in respiratory rate, tidal volume, and even the recruitment of accessory respiratory muscles. Consequently, these changes may affect the overall efficiency of pulmonary gas exchange, potentially leading to suboptimal respiratory function [5].

Septoplasty is one of the most frequently performed surgeries in the otorhinolaryngology setting. The various indications for septoplasty are nasal obstruction, nasal crusting, rhinorrhea, post-nasal discharge, recurrent sinus pressure or pain, epistaxis, headache, excessive snoring, and sleep apnea. The Sino-Nasal Outcome Test-22 (SNOT-22) questionnaire recorded preoperatively is very useful in knowing the severity of the patient’s nasal symptoms and helps in the selection of patients for septoplasty. Additionally, the postoperative SNOT-22 questionnaire helps in comprehending the outcome regarding the efficacy of the surgery. It also assists in analyzing the severity of symptoms in everyday clinical practice [6].

Because of the nasal obstruction seen in NSD, dry and cold air and other particles may gain entry into the lower airway and may lead to bronchoconstriction and lower airway irritation. Therefore, there seems to be a significant relationship between the upper and lower airways [7].

Comprehending the potential enhancements in pulmonary function after septoplasty is paramount for optimizing patient outcomes and for understanding the broader interactions between upper and lower respiratory physiology. By relieving the web of interactions between the nasal and pulmonary systems, our study aims to potentially guide clinical practices and highlight the benefits of addressing NSD [8].

Our main goal is to evaluate the enhancement in PFTs following septoplasty in individuals with a deviation of the nasal septum.

## Materials and methods

This longitudinal study was designed to assess the influence of septoplasty on PFTs in patients diagnosed with a deviated nasal septum (DNS) in the Department of Otolaryngology and Head and Neck Surgery. Data was collected from all patients visiting the Ear, Nose, and Throat (ENT) Outpatient Department (OPD) at a tertiary care center who presented with nasal obstruction and were clinically diagnosed with DNS. The study was conducted over an 18-month period, from October 1, 2022, to March 31, 2024.

Patients aged 18-55 years, both male and female, with nasal obstruction due to isolated DNS, who consented to undergo septoplasty were included in the study. Patients younger than 18 years or older than 55 years, patients undergoing septoplasty in combination with sinus or other nasal surgeries, or patients with comorbidities such as hypertension, coronary artery disease, diabetes mellitus, chronic smoking, chronic obstructive pulmonary disease (COPD), bronchial asthma, excessive turbinate hypertrophy, chronic sinusitis, or nasal polyposis were excluded from the study.

The sample size was calculated from the data and results of a similar study conducted by Tuzuner et al. (2016) [9], where, considering the 50% of forced vital capacity (FIF50) pre-operatively and post-operatively as 3.0 ± 1.1 and 4.6 ± 1.2, respectively, with a confidence interval (CI) of 95% and a power of 80%, the minimum sample size calculated was 20. However, in this study, we have included 60 participants. The software used is WinPepi (PEPI-for-Windows) version 11.38.

Institute Ethics Committee clearance was obtained with IESC/PGS/2022/124 before initiating the study. Informed consent was obtained from the participants. A detailed history and ear, nose, and throat examination were conducted. Routine blood investigations and X-rays of the chest and paranasal sinuses (Waters' view) were performed. PFTs, including spirometry and the six-minute walk test (6MWT), were conducted pre-operatively. Patients completed the SNOT22 questionnaire prior to surgery. Septoplasty was performed on the selected patients. At three weeks post-operatively, spirometry and a 6MWT were repeated. Patients also completed the SNOT22 questionnaire post-surgery to assess changes in symptom severity [6].

Data analysis

The collected data were analyzed to assess how septoplasty affects pulmonary function and alleviates symptoms. Pre- and post-operative PFT values and SNOT22 scores were compared to determine the extent of improvement. Statistical analysis was conducted to assess the significance of the changes observed using IBM SPSS Statistics for Windows, Version 24 (Released 2016; IBM Corp., Armonk, NY, USA). Descriptive statistics summarized demographics, baseline characteristics, and preoperative SNOT22 scores. Paired t-tests and Wilcoxon signed-rank tests compared pre- and post-operative means of continuous variables, with significance set at p < 0.05. Repeated measures analysis of variance (ANOVA) assessed changes in PFT values and 6MWT distances over time, examining interaction effects with demographic variables. Changes in SNOT22 scores were analyzed similarly. Pearson correlation coefficients explored relationships between changes in PFT values and SNOT22 scores, while multiple linear regression identified predictors of post-operative improvement. Stratified and sensitivity analyses controlled for confounders and assessed result stability. Results were presented in tables and figures, with means, standard deviations, CIs, and p-values, and interpreted in the context of clinical relevance and existing literature.

## Results

Out of the 60 patients in our study diagnosed with DNS, 30 (50%) were males. The mean age of the participants was 35.6 ± 8.2 years. This distribution indicates a diverse age range among the participants, allowing for a comprehensive assessment of septoplasty outcomes across different age groups.

Comparing pre-operative and post-operative SNOT22 scores reveals significant improvements across all symptom categories following the procedure. Nasal congestion, facial pain/pressure, and nasal discharge exhibit substantial reductions in mean scores, decreasing from 4.6 ± 0.3, 4.4 ± 0.4, and 4.5 ± 0.4, respectively, pre-operatively to 2.2 ± 0.5, 2.0 ± 0.3, and 2.3 ± 0.4 post-operatively, all with p-values < 0.001. Loss of smell/taste also sees improvement, albeit with a slightly higher p-value of < 0.01, decreasing from 3.6 ± 1.8 pre-operatively to 1.8 ± 1.0 post-operatively; therefore, it is relatively less significant than the other parameters. These findings indicate the efficacy of the procedures in alleviating symptoms associated with the patients' conditions (Table 1).

**Table 1:** Comparison of pre and postoperative symptoms severity with Sinonasal Outcome Test (SNOT22) questionnaire Values are represented as mean ± SD, and comparisons were made using a one-way ANOVA test, with p < 0.001 considered statistically significant. ANOVA: Analysis of variance

Symptoms	Preoperative (n = 60)	Postoperative (n = 60)	p-value
Nasal congestion	4.6 ± 0.3	2.2 ± 0.5	<0.001
Facial pain/pressure	4.4 ± 0.4	2.0 ± 0.3	<0.001
Nasal discharge	4.5 ± 0.4	2.3 ± 0.4	<0.001
Loss of smell/taste	3.6 ± 1.8	1.8 ± 1.0	<0.01

The comparison between pre-operative and post-operative pulmonary function parameters highlights statistically significant improvements across all metrics following the procedure. Forced expiratory volume in one second (FEV1) increases from 85.4% ± 7.2 pre-operatively to 88.2% ± 6.5 post-operatively, alongside an elevation in forced vital capacity (FVC) from 87.9% ± 6.8 to 90.5% ± 6.0. The FEV1/FVC ratio improves from 0.78 ± 0.05 to 0.80 ± 0.04, and peak expiratory flow rises from 380 ± 40 mL/s to 390 ± 35 mL/s, all with p-values < 0.05. These findings underscore the effectiveness of the procedure in enhancing pulmonary function among the patients (Table 2).

**Table 2:** Comparison of pre and postoperative pulmonary function test results Values are represented as mean ± SD, and comparisons were made using a one-way ANOVA test, with p < 0.05 considered statistically significant. FEV1: Forced expiratory volume in one second; FVC: Forced vital capacity; ANOVA: Analysis of variance

Parameter	Preoperative values (n = 60)	Postoperative values (n = 60)	p-value
FEV1 (%)	85.4 ± 7.2	88.2 ± 6.5	<0.05
FVC (%)	87.9 ± 6.8	90.5 ± 6.0	<0.05
FEV1/FVC	0.78 ± 0.05	0.80 ± 0.04	<0.05
Peak expiratory flow	380 ± 40 mL/s	390 ± 35 mL/s	<0.05

The comparison of pre-operative and post-operative physical parameters demonstrates statistically significant improvements across all metrics following the procedure. The average distance walked increases from 425 ± 30 meters pre-operatively to 460 ± 40 meters post-operatively, while the heart rate decreases from 85 ± 10 bpm to 80 ± 8 bpm. Additionally, oxygen saturation levels rise from 98% ± 1 to 99% ± 1, with all changes showing p-values < 0.05. These findings indicate enhanced physical fitness and cardiovascular health among the patients after their interventions (Table 3).

**Table 3:** Comparison of pre and postoperative exercise capacity using a six-minute walk test (6MWT) Values are represented as mean ± SD, and comparisons were made using a one-way ANOVA test, with p < 0.05 considered statistically significant. ANOVA: Analysis of variance

Parameter	Preoperative (n = 60)	Postoperative (n = 60)	p-value
Distance walked (meters)	425 ± 30	460 ± 40	<0.05
Heart rate (beats per minute)	85 ± 10	80 ± 8	<0.05
Oxygen saturation (%)	98 ± 1	99 ± 1	<0.05

**Figure 1:**
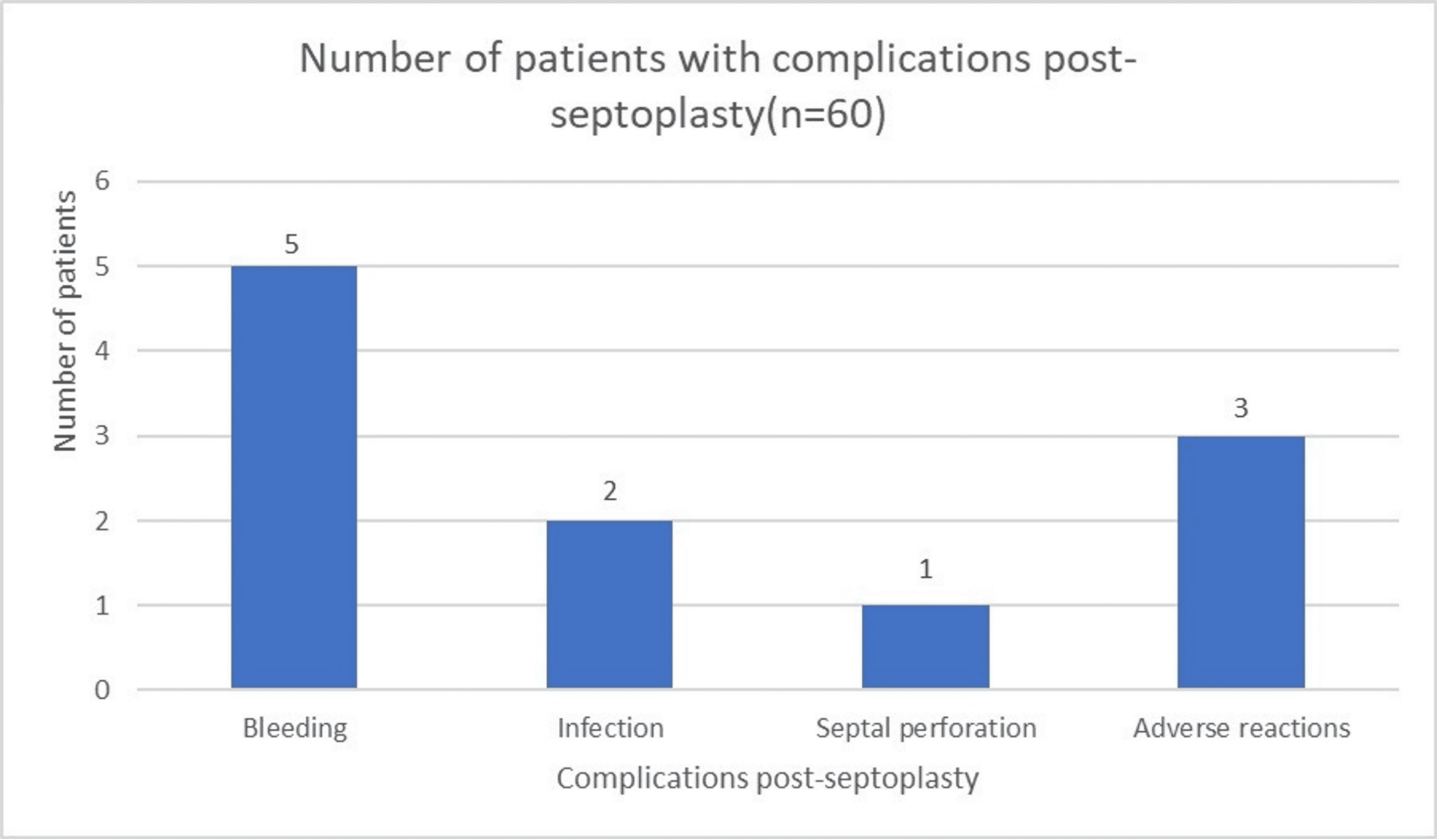
Complications post-septoplasty

Among the 60 patients, complications post-operatively were relatively infrequent, with only a small number experiencing adverse events. Specifically, five patients encountered bleeding, two faced infections, one developed septal perforation, and three experienced adverse reactions. These findings underscore the importance of comprehensive post-operative monitoring and management to promptly address and minimize complications, ensuring optimal patient recovery and well-being following the procedures (Figure 1).

Among the 60 patients, 35 (58.3%) were very satisfied with the surgery, and 20 (33.3%) were satisfied. Three (5%) patients were neutral regarding the surgery, two (3.3%) were dissatisfied, while no one was very dissatisfied. These results suggest that the majority of patients experienced positive outcomes and were content with the interventions, highlighting the effectiveness of the procedures in meeting their expectations and improving their quality of life.

The hospital stay duration for the 60 patients undergoing the procedures is characterized by a mean of 2.5 days, with a standard deviation of 0.8 days.

## Discussion

Septoplasty aims to restore normal nasal airflow, which is hypothesized to enhance pulmonary function. Our study evaluates the influence of septoplasty on PFTs, crucial indicators of respiratory health. Improvement in PFTs post-septoplasty could demonstrate the broader benefits of this procedure beyond merely alleviating nasal obstruction, potentially offering enhanced overall respiratory function and quality of life for patients [10,11]. Septoplasty, a surgical intervention performed to correct a DNS, is primarily conducted to alleviate nasal obstruction and enhance airflow through the nasal passages. NSD can significantly impair respiratory function, contributing to chronic nasal congestion, sinusitis, and sleep disturbances such as obstructive sleep apnea [12,13].

Our study involved 60 patients with a mean age of 35.6 ± 8.2 years, equally divided between 30 males and 30 females. Singh et al. (2022) included 50 patients aged 18-50, with a demographic composition of 78% males and 22% females [14]. Yigit et al. (2022) assessed 30 patients with a mean age of 33.7 ± 10.9 years, of whom 56.7% were males [15]. In contrast, Dankert et al. (2022) conducted a systematic review of 46 original studies, encompassing a varied participant demographic across the included research [16].

Pre-operative symptom severity was evaluated using the SNOT22 score, which revealed that nasal congestion, facial pain/pressure, and nasal discharge were the most severe symptoms, with mean scores of 4.6 ± 0.3, 4.4 ± 0.4, and 4.5 ± 0.4, respectively. Loss of smell/taste had a mean score of 3.6 ± 1.8, indicating wider variability. These findings highlight the significant burden of symptoms experienced by patients with DNS, justifying the need for effective surgical intervention. The high severity scores also provide a robust baseline against which post-operative improvements can be measured. In our study, the clinical presentation included nasal congestion, facial pain or pressure, nasal discharge, and loss of smell or taste. Singh et al. (2022) reported symptoms of nasal obstruction, sneezing, nasal discharge, and headache [14]. Yigit et al. (2022) focused on symptoms associated with NSD [15]. In contrast, Dankert et al. (2022) did not specify clinical presentations, as their study was a systematic review of existing literature [16].

The SNOT-22 is a validated patient-reported outcome measure designed to evaluate the impact of chronic rhinosinusitis on a patient's quality of life. It consists of 22 questions that assess both physical symptoms and emotional consequences associated with sinonasal disorders. Patients rate each item on a scale from 0 (no problem) to 5 (problem as bad as it can be), yielding total scores ranging from 0 to 110. Key areas covered include nasal blockage, facial pain, sleep disturbance, and emotional well-being. The SNOT-22 is widely used in clinical practice and research to quantify symptom severity, aid in treatment decisions, and monitor treatment effectiveness. Its comprehensive assessment allows clinicians to understand the diverse ways chronic rhinosinusitis affects patients' daily lives and to develop personalized treatment plans accordingly.

The pre-operative PFT results showed a mean FEV1 of 85.4% ± 7.2, a mean FVC of 87.9% ± 6.8, and a mean FEV1/FVC ratio of 0.78 ± 0.05. The mean peak expiratory flow was 380 ± 40 mL/s. These results indicate that, while patients had generally acceptable levels of lung function, there was variability that could potentially be improved with septoplasty.

Exercise capacity, assessed through the 6MWT, revealed a mean distance of 425 ± 30 meters, a mean heart rate of 85 ± 10 bpm, and a mean oxygen saturation of 98% ± 1. These findings provide a baseline of the patients' functional exercise capacity and cardiopulmonary status, highlighting areas for potential improvement post-surgery.

Three weeks post-septoplasty, significant improvements were observed in pulmonary function. The mean FEV1 increased to 88.2% ± 6.5, the mean FVC rose to 90.5% ± 6.0, and the mean FEV1/FVC ratio improved to 0.80 ± 0.04. Additionally, the mean peak expiratory flow increased to 390 ± 35 mL/s. These enhancements in pulmonary function metrics indicate that septoplasty had a positive impact on the respiratory mechanics of the patients.

In terms of exercise capacity, the 6MWT showed an increase in the mean distance walked to 460 ± 40 meters. The mean heart rate decreased to 80 ± 8 bpm, and the mean oxygen saturation improved to 99% ± 1. These improvements suggest enhanced exercise tolerance and better cardiopulmonary efficiency, likely resulting from reduced nasal obstruction and improved airway patency post-surgery.

Post-operative SNOT22 scores revealed substantial reductions in symptom severity. Nasal congestion, facial pain/pressure, and nasal discharge showed significant decreases, with mean scores dropping to 2.2 ± 0.5, 2.0 ± 0.3, and 2.3 ± 0.4, respectively, all with p-values < 0.001. Loss of smell/taste also improved, with scores reducing to 1.8 ± 1.0 (p-value < 0.01). These findings highlight the efficacy of septoplasty in alleviating the primary symptoms of DNS, thereby significantly improving patient comfort and quality of life.

Singh et al. (2022) in their postoperative assessment, revealed improvements in PFT values across all patients, with statistically significant enhancements observed in FEF25% and FIF75% (p < 0.05), particularly in patients with type II DNS. The study concluded that septoplasty positively impacts PFT values, underscoring the influence of a DNS on lower airway function [14].

Yigit et al. (2022), in their post-operative assessment, reported improvements in PFT values following septoplasty, indicating a positive impact on lower airway function. However, they may have observed variations in the statistical significance of improvements across different PFT parameters compared to Singh et al. (2022). Despite similarities in objectives and patient demographics, the studies underscore the importance of consulting multiple sources to gain a comprehensive understanding of how septoplasty influences pulmonary function [15].

In contrast, Dankert et al. (2022) did not specify improvement in PFT values, as their study was a systematic review of existing literature. They conducted a systematic review to assess the effectiveness of PFTs, specifically spirometry and blood gas analysis, in improving preoperative risk assessment for non-thoracic surgery. The review involved a thorough search of databases such as MEDLINE, CINAHL, and the Cochrane Library, following predefined criteria and PRISMA-DTA guidelines. The qualitative synthesis of prospective studies provided inconclusive results: 65% of studies supported the use of preoperative spirometry, while 35% did not. Regarding blood gas analysis, 43% of studies supported its utility [16].

In our study, the post-operative complications were relatively infrequent, with only a few patients experiencing adverse events: five had bleeding, two faced infections, one developed septal perforation, and three experienced adverse reactions. These findings highlight the importance of comprehensive post-operative monitoring and management to promptly address and minimize complications, ensuring optimal patient recovery and well-being following the procedures. The mean hospital stay duration was 2.5 days, with a standard deviation of 0.8 days, ranging from one to five days. This relatively short hospital stay indicates efficient management and recovery protocols, allowing most patients to be discharged within a few days of their procedures.

The results of this study have several important clinical implications. First, the significant improvements in pulmonary function and exercise capacity post-septoplasty suggest that correcting nasal structural anomalies can have broader respiratory benefits beyond just alleviating nasal obstruction. This is particularly relevant for patients who may have underlying or concomitant lower airway conditions that could be exacerbated by impaired nasal airflow. Second, the substantial reductions in SNOT22 scores post-operatively highlight the value of septoplasty in significantly alleviating the subjective symptom burden associated with DNS. This is crucial for patient-centered care, as improved symptomatology directly translates to enhanced quality of life and patient satisfaction. Third, the gender-balanced and age-diverse sample enhances the generalizability of the findings, suggesting that septoplasty can be beneficial across a wide demographic spectrum. This information is crucial for guiding clinical decision-making and effectively counseling patients, ensuring that the advantages of septoplasty are clearly communicated to a wide range of patients.

Our study holds significant implications for both clinical practice and the broader understanding of respiratory physiology. By considering the potential improvements in pulmonary function following septoplasty, clinicians can better counsel patients regarding the holistic benefits of surgical intervention [17].

While we attempted to perform a comprehensive analysis of the effects of septoplasty on PFTs, it is bound to have some limitations. The study's sample size of 60 patients may not be sufficient to generalize the findings to a larger population, and data collected solely from a single tertiary care center may restrict the external validity and generalizability of the findings to other populations or settings.

## Conclusions

Our study demonstrates that septoplasty is an effective surgical intervention for improving pulmonary function, exercise capacity, and symptom severity in patients with deviated nasal septa. The significant improvements observed in PFTs, 6MWT results, and SNOT22 scores post-operatively highlight the procedure's ability to enhance respiratory health and overall quality of life. The findings are applicable across a diverse age and gender spectrum, underscoring the broad utility of septoplasty. Long-term follow-up studies would be valuable to assess the durability of the improvements observed in pulmonary function. These results provide a strong foundation for further research into the long-term benefits and mechanisms of septoplasty, paving the way for more refined and effective treatment strategies for patients with nasal structural anomalies.
